# Supplementary material for: Interpretable multimodal machine learning (IMML) framework reveals pathological signatures of distal sensorimotor polyneuropathy
Source: Commun Med (Lond). 2024 Dec 16;4:265. doi: 10.1038/s43856-024-00637-1 (PMC11649904; doi:10.1038/s43856-024-00637-1)
Supplement: Supplementary file 2 — Supplementary Information [file 43856_2024_637_MOESM2_ESM.pdf]

Supplementary Information

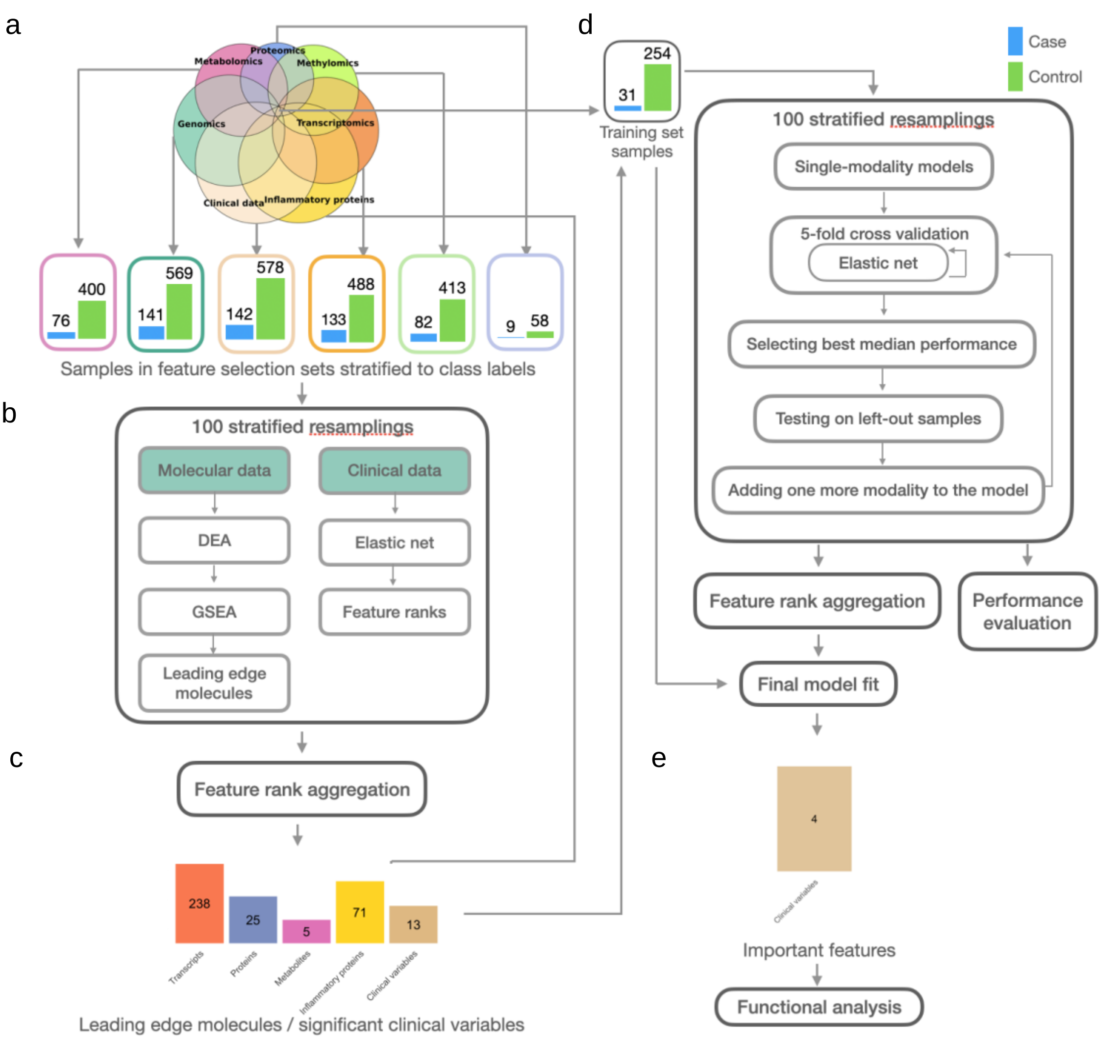

Supplementary Figure S1. Analysis pipeline and results for cross-sectional DSPN

a. Features in modality-specific datasets were selected independently using non-overlapping modality-specific samples. The selected features stratified into case and control are shown in the barplots.
b. Molecular data went through differential expression analysis (DEA) which generated a molecule list sorted by t-statistics which was then used as input for gene set enrichment analysis (GSEA). GSEA output leading edge genes which drive the enrichment of their respective gene sets. Clinical features was selected by training elastic net models and extracting important features. The process was repeated using 100 stratified resamplings.
c. The final significant list of molecules and clinical variables were selected using a rank aggregation algorithm.
d. After feature selection step, the selected features were then integrated to train models to predict DSPN, using the left-out overlapping dataset (training set). The training aimed to determine the optimal complexity and composition of the models by implementing elastic net with forward feature selection in a nested cross-validation manner, using weighted log loss as performance metric to account for class imbalance. We used 100 stratified resamplings during training and the rank aggregation at the end to select the most stable model.

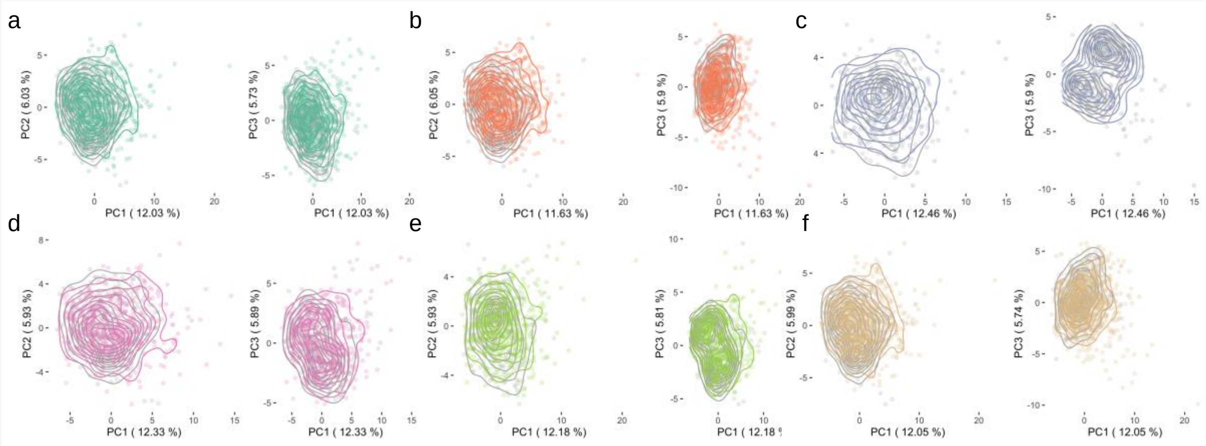

Supplementary Figure S2. PCA of clinical features for feature selection and model training datasets. Grey contour plots highlight the model training sets, whilst other colors indicate the feature selection set of the different modalities: (a) Genomics, (b) Transcriptomics, c) Proteomics, (d) Metabolomics, (e) Methylomics and (f) Clinical data.

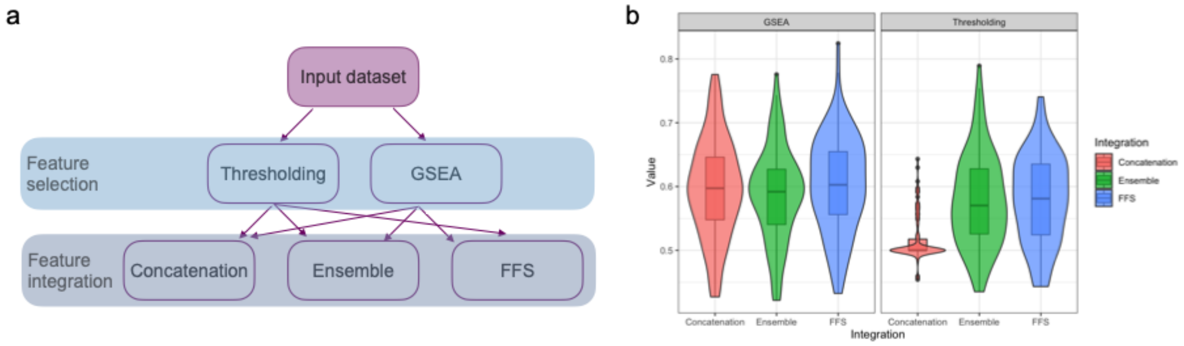

Supplementary Figure S3. Benchmarking of feature selection and integration methods

a. Illustration of methods for feature selection (thresholding and GSEA) and feature integration (concatenation, ensemble and our FFS algorithm) in a conventional multi-modal machine learning process. Arrows show the possible trajectory of the process in which different combinations of these methods could be used.
b. Benchmarking result showing prediction performance on the test set of different selection-integration methods for incident DSPN prediction using transcriptomic, proteomic, metabolomic and clinical data. Distributions of AUROC for the matched 100 stratified resamplings are shown in the y-axis and different methods are shown on the x-axis.

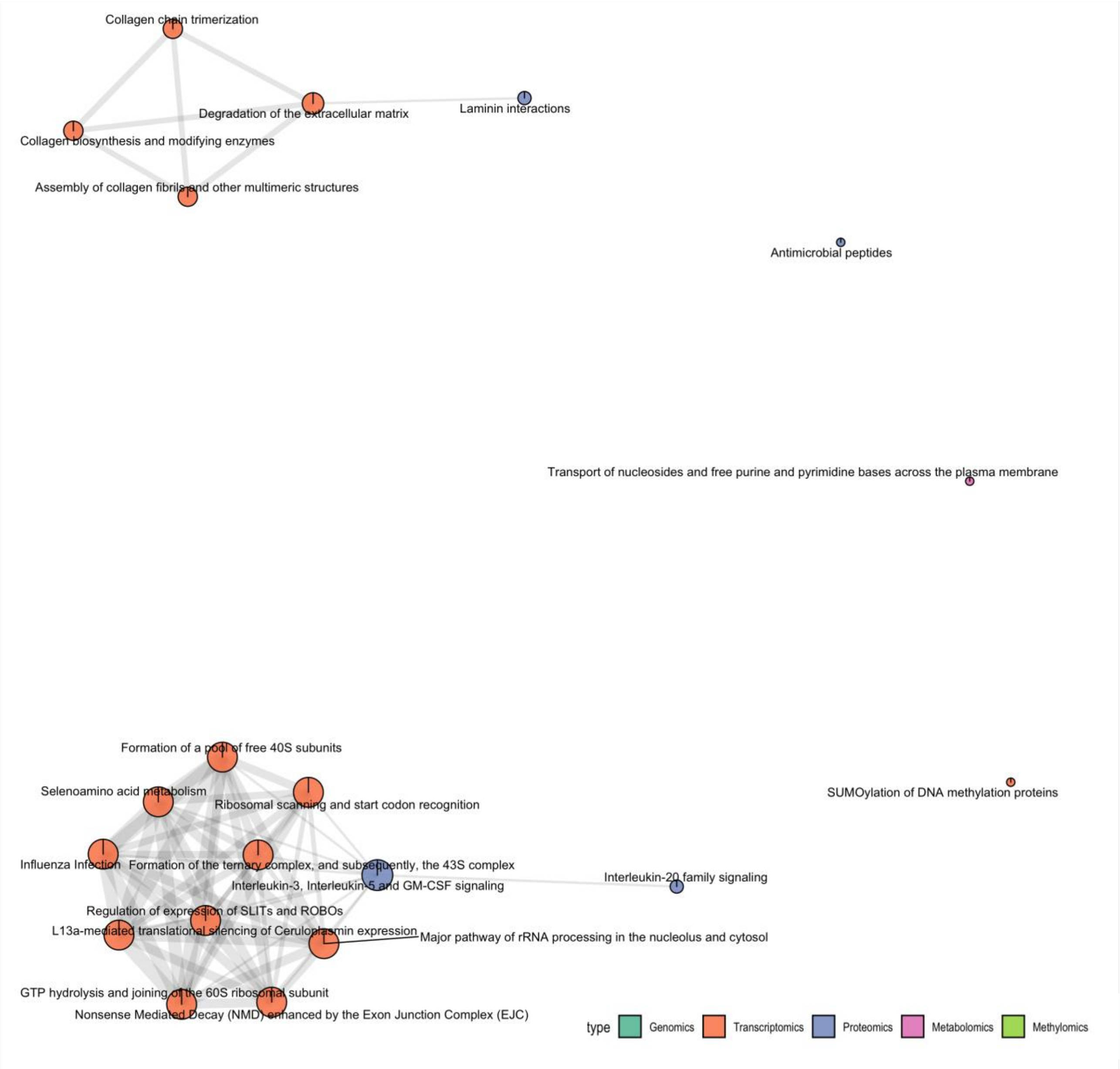

Supplementary Figure S4. Network of enriched gene sets in cross-sectional DSPN

Network of enriched gene sets from which the predictive features were selected, for cross-sectional DSPN prediction. Nodes are the gene sets coloured with their corresponding data modality. Size of the nodes reflects their centrality with respect to the network. Edges are the number of shared leading-edge molecules between two nodes.

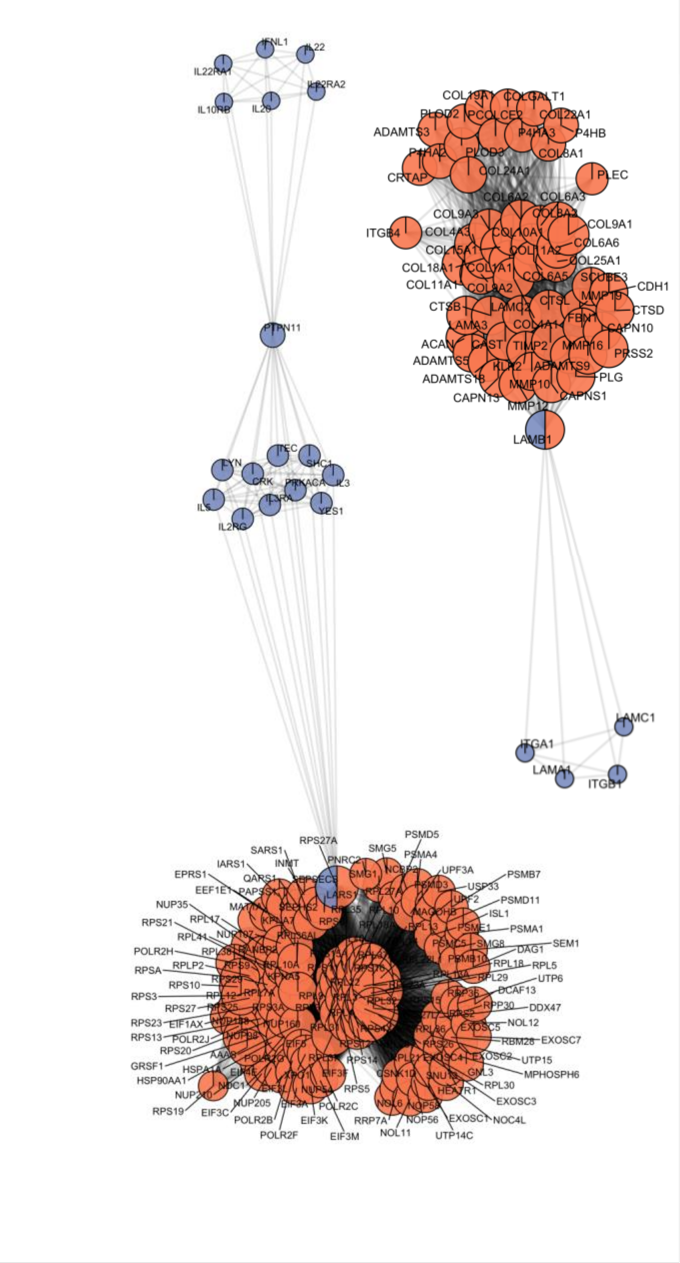

Supplementary Figure S5. Network of enriched features in cross-sectional DSPN

Network of all selected features for training cross-sectional DSPN models. Nodes are the features coloured with their corresponding data modality. Edges are the number of shared gene sets between two nodes.

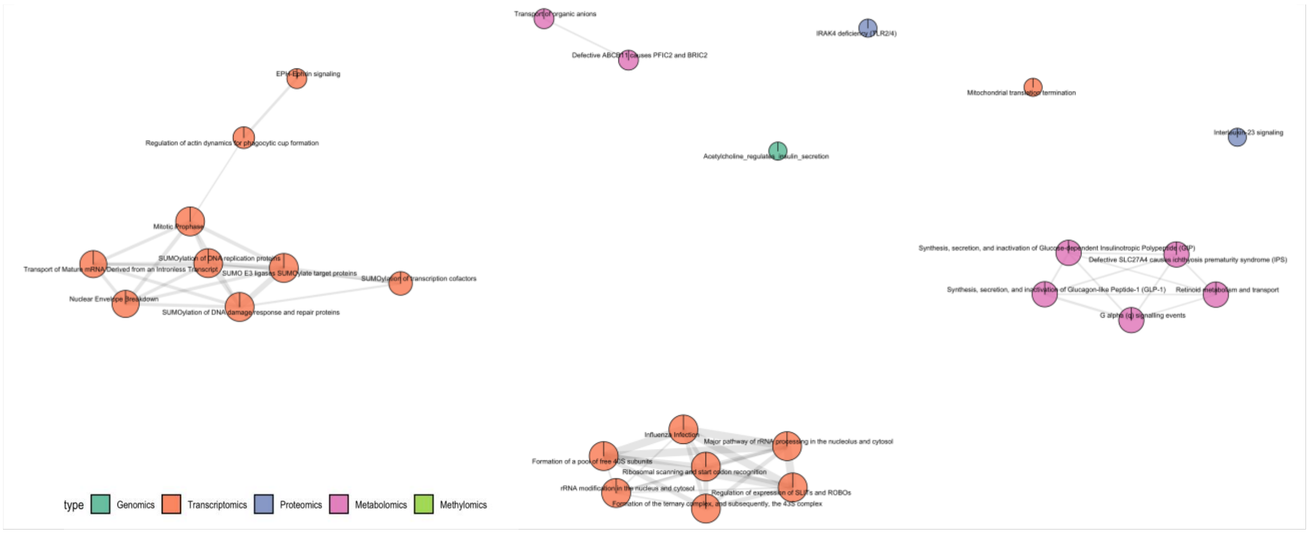

Supplementary Figure S6. Network of enriched gene sets in incident DSPN
Network of enriched gene sets from which the predictive features were selected, for incident DSPN prediction. Nodes are the gene sets coloured with their corresponding data modality. Size of the nodes reflects their centrality with respect to the network. Edges are the number of shared leading-edge molecules between two nodes.

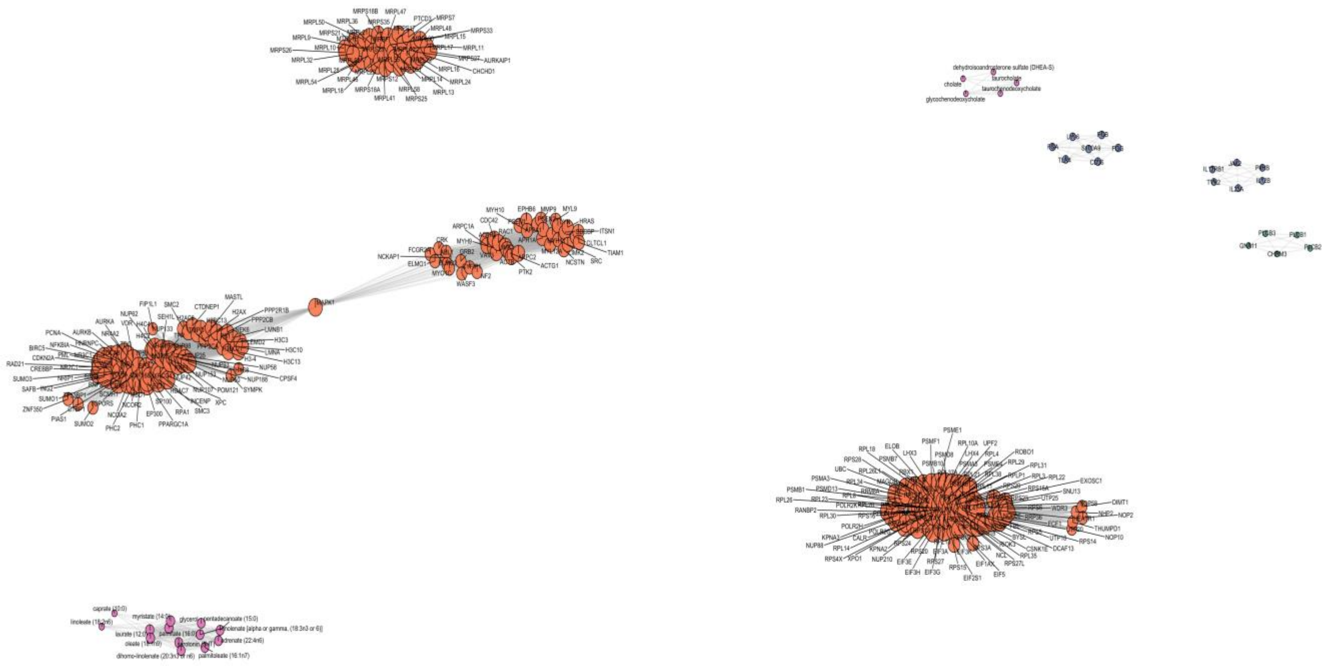

Supplementary Figure S7. Network of enriched features in incident DSPN
Network of all selected features for training incident DSPN models. Nodes are the features coloured with their corresponding data modality. Edges are the number of shared gene sets between two nodes.

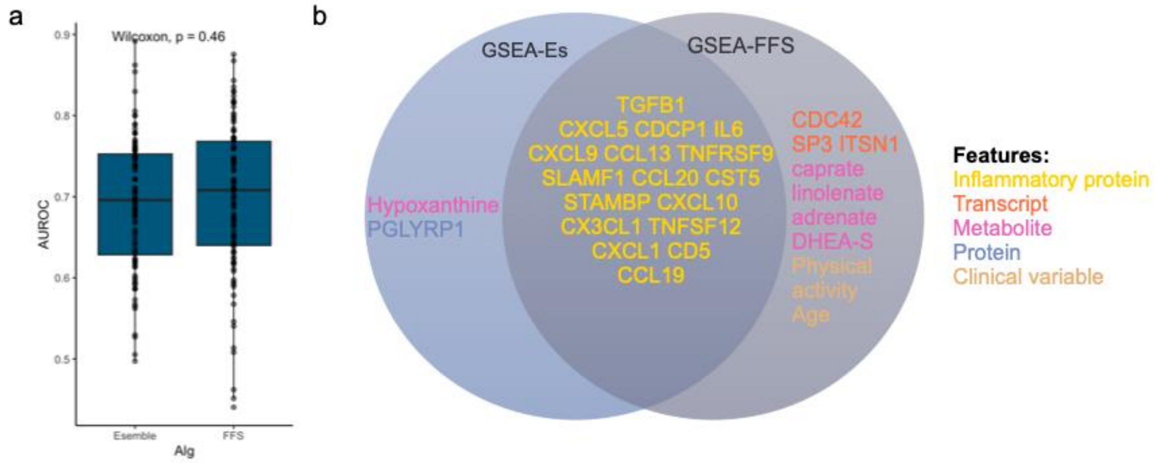

Supplementary Figure S8. Performance of forward feature selection (FFS) and ensemble stacking feature integration methods across 100 stratified resamples. (a) AUROC of the testing prediction of the two algorithms. P-value of Wilcoxon rank sum test is shown.(b) Important features selected by the GSEA-ensemble stacking (GSEA-Es) and GSEA-FFS methods and their overlapping.

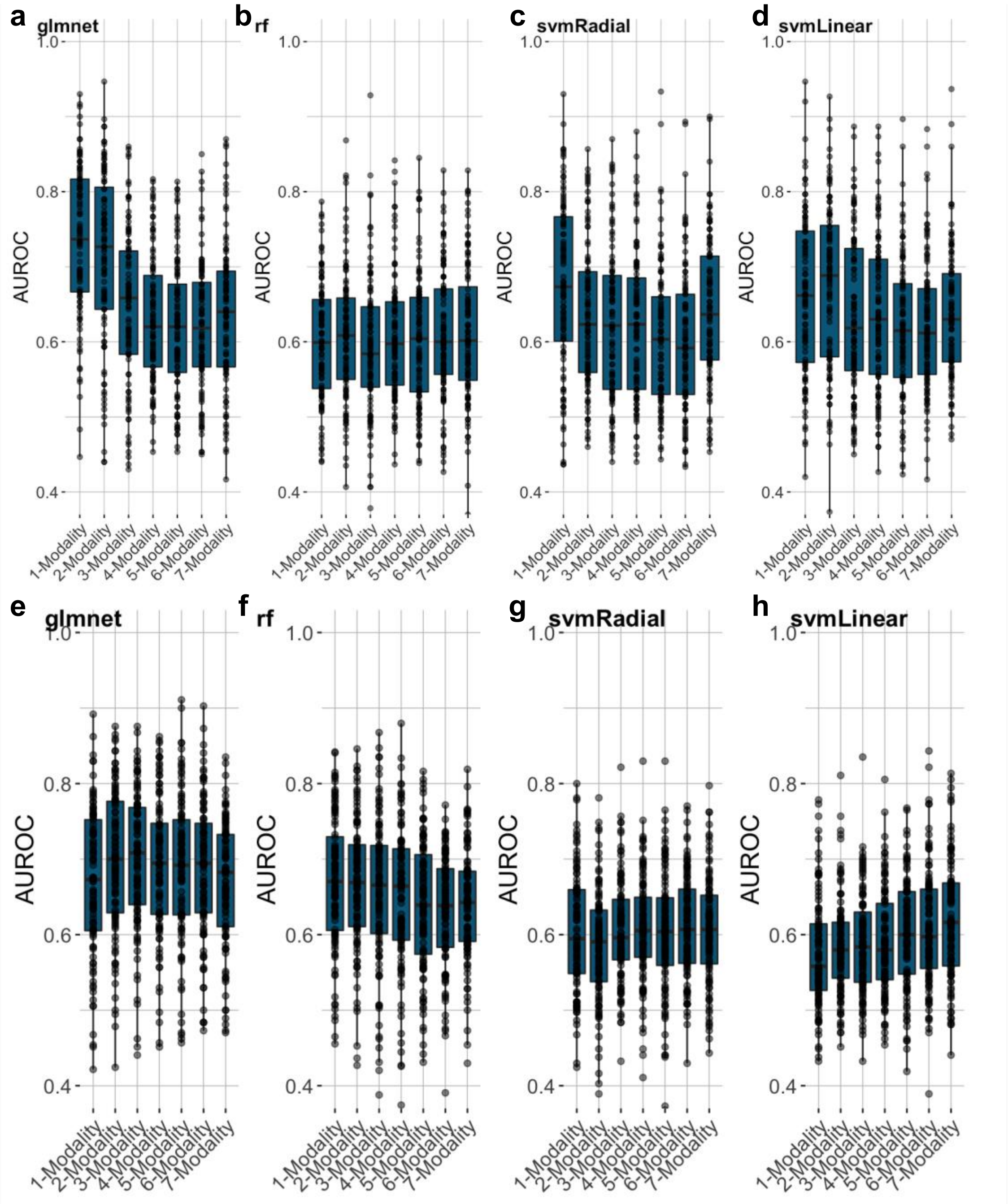

Supplementary Figure S9: Prediction performance of four different machine learning algorithms. Here we compare the predictive power of (a-d) prevalent DSPN and (e-h) incident DSPN. We benchmarked (a,e) elastic net (glmnet), (b,f) random forest (rf), and support vector machine with (c,g) radial (svmRadial) and (d,h) linear kernel (svmLinear).

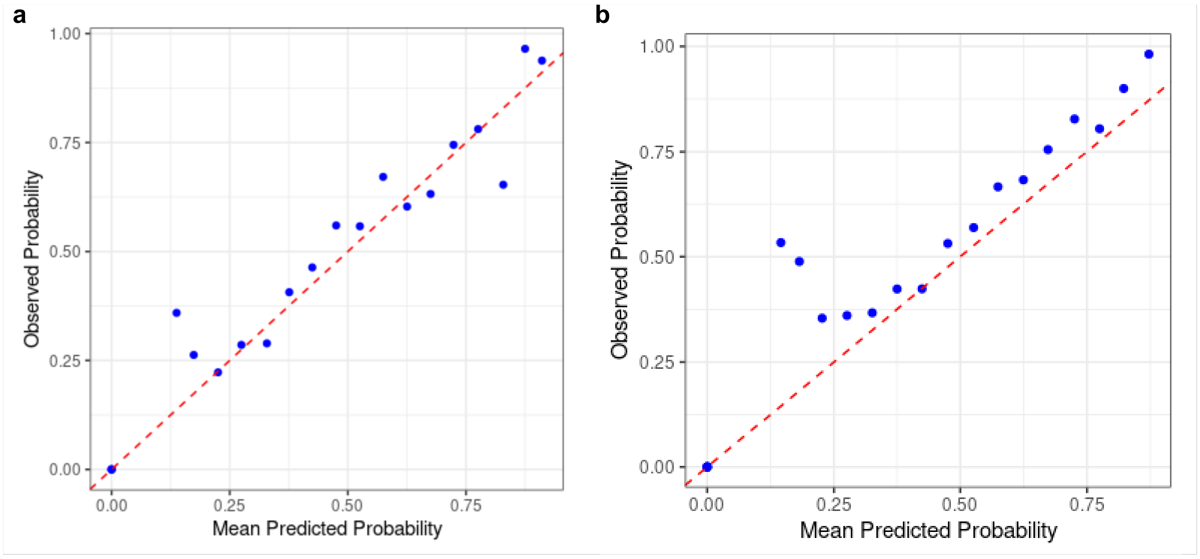

Supplementary Figure S10: Calibration plots of predicted probabilities for prevalent DSPN (a) and incident DSPN (b). The predicted probabilities were calibrated using the Platt scaling method.

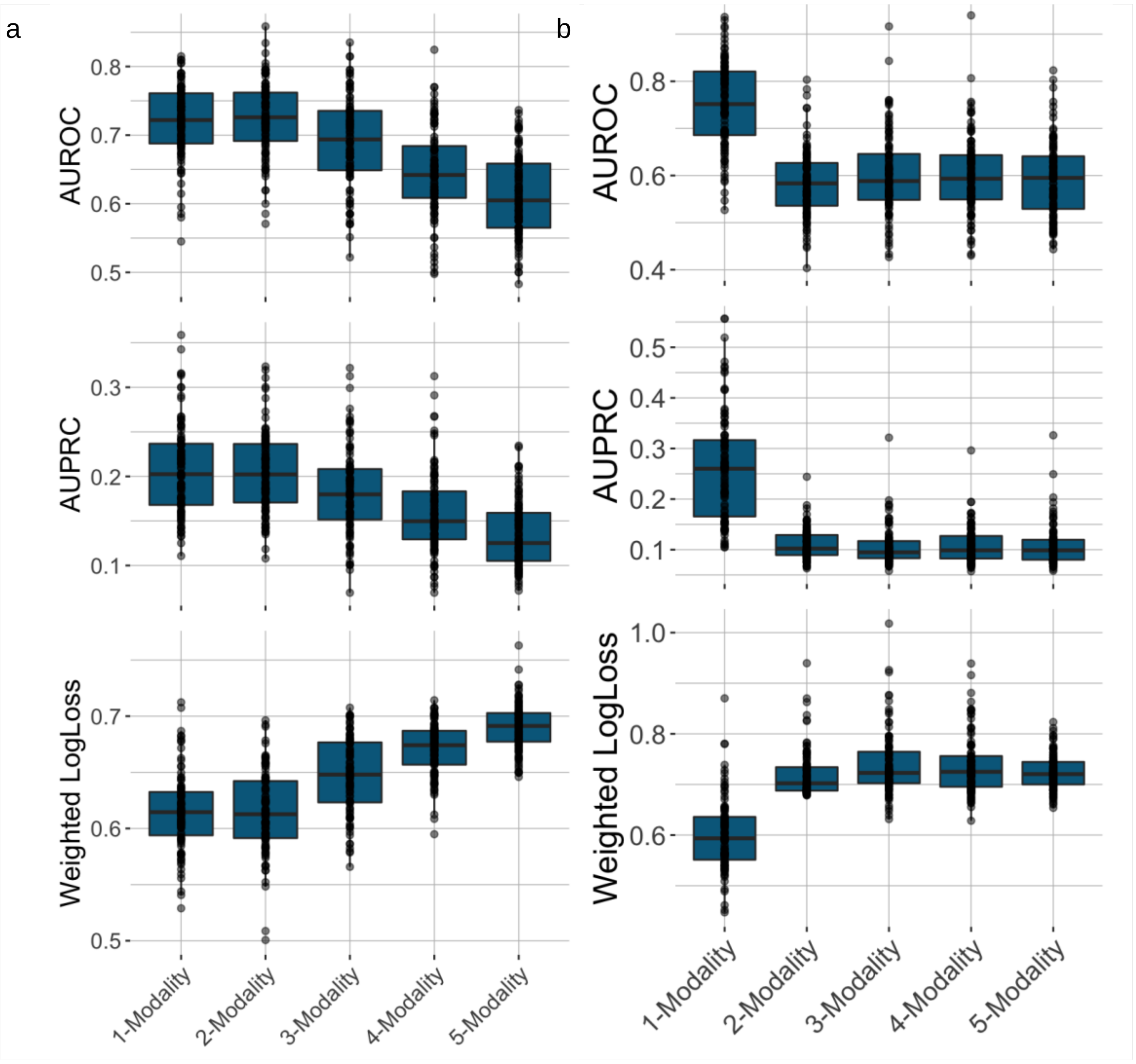

Supplementary Figure S11. Prediction performance of prevalent DSPN models, when forcing the FFS algorithm to choose clinical model at the beginning.

a. Prediction performance during cross-validation. X-axis shows the increasing model complexity. Y-axis shows the median of performance values across 5-fold cross-validation for AUROC, AUPRC and weighted log-loss
b. Prediction performance on the testing sets. X-axis shows the increasing model complexity. Y-axis shows the performance values on the testing sets for AUROC, AUPRC and weighted log-loss

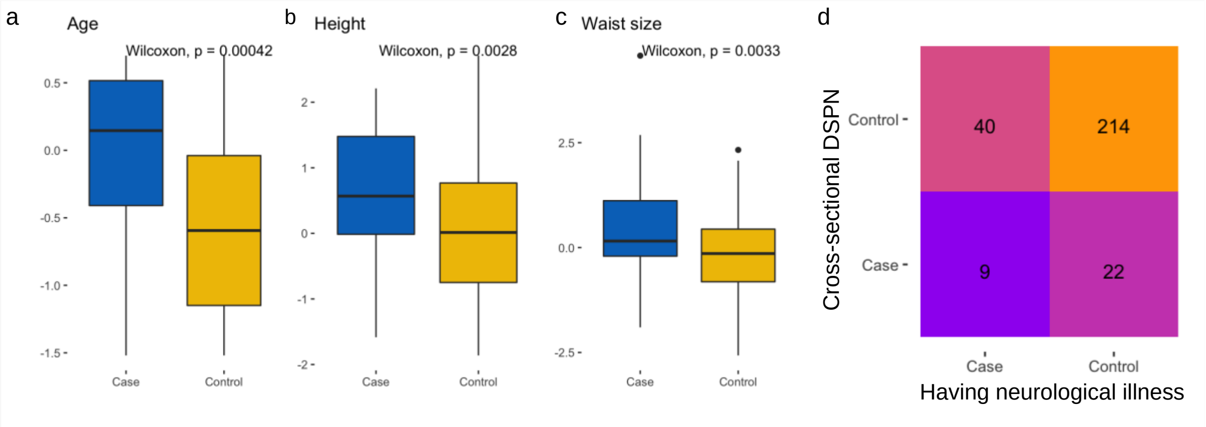

Supplementary Figure S12. Distribution of important clinical variables for cross-sectional DSPN model

Distribution of age, height and waist size in the training set stratified into case and control (panel a, b and c respectively). Panel d shows association of patients who have neurological illness in general and cross-sectional DSPN. P-values for Wilcoxon rank sum test and Fisher's exact test are shown.

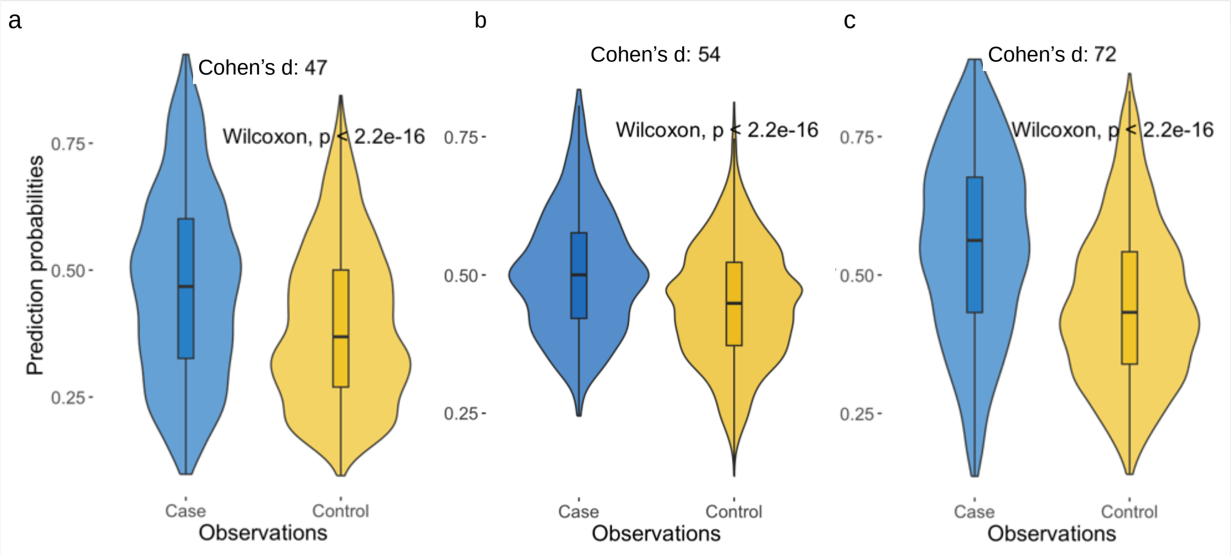

Supplemental Figure S13: Baseline models to predict DSPN incidence. Prediction probabilities during testing of negative samples using a) the prevalent DSPN model trained on clinical data alone at F4, b) baseline incidence model trained only on clinical variables at F4 and incidence label at FF4 and c) the full incidence model trained on clinical + molecular variables at F4 and incidence label at FF4. Cases are samples developing DSPN from F4 to FF4, and controls are ones remaining negative. For each comparison, Cohen's d was used as the measure of the difference between groups.

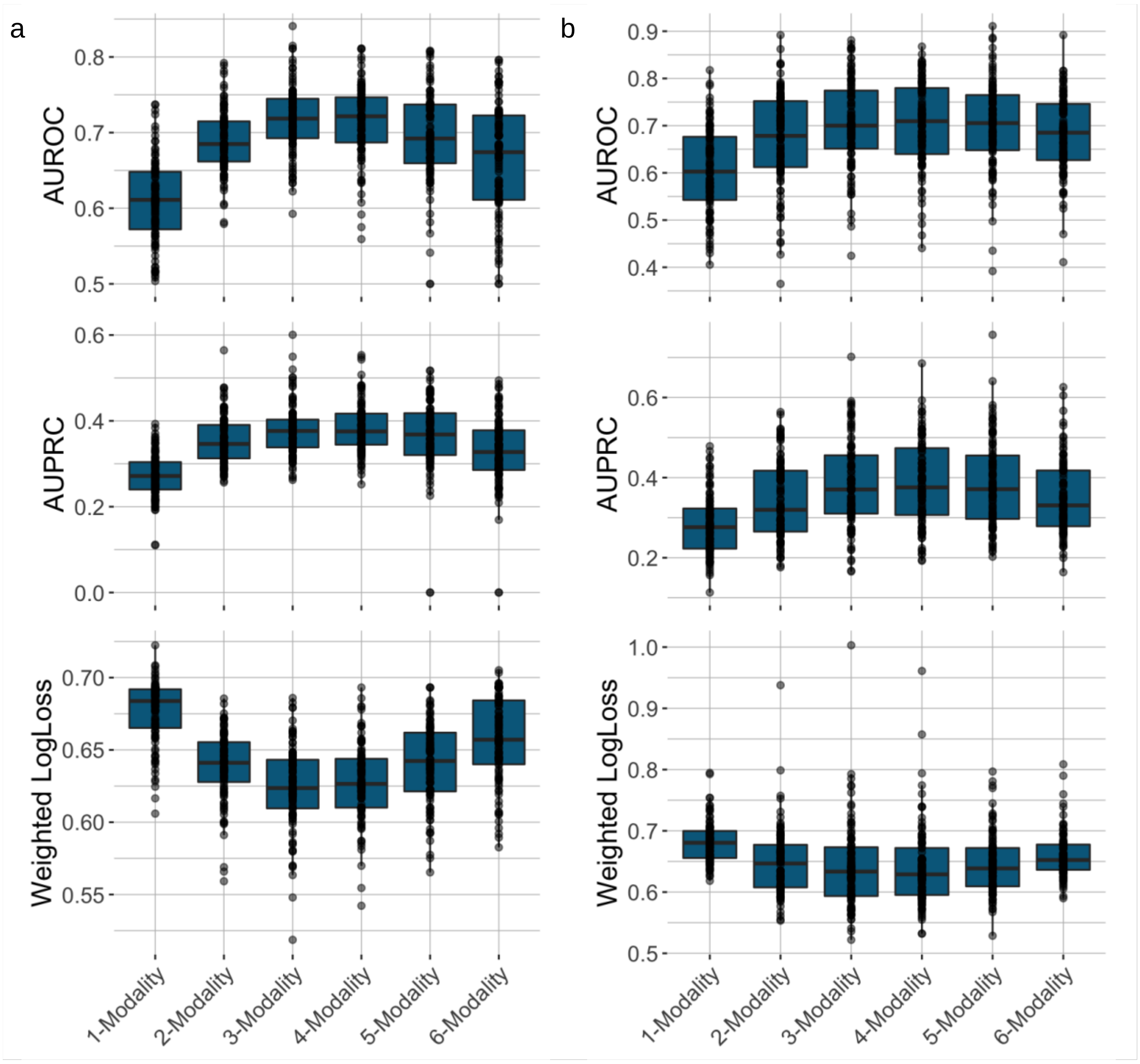

Supplementary Figure S14. Prediction performance of incident DSPN models, when forcing the FFS algorithm to choose clinical model at the beginning.

a. Prediction performance during cross-validation. X-axis shows the increasing model complexity. Y-axis shows the median of performance values across 5-fold cross-validation for AUROC, AUPRC and weighted log-loss
b. Prediction performance on the testing sets. X-axis shows the increasing model complexity. Y-axis shows the performance values on the testing sets for AUROC, AUPRC and weighted log-loss

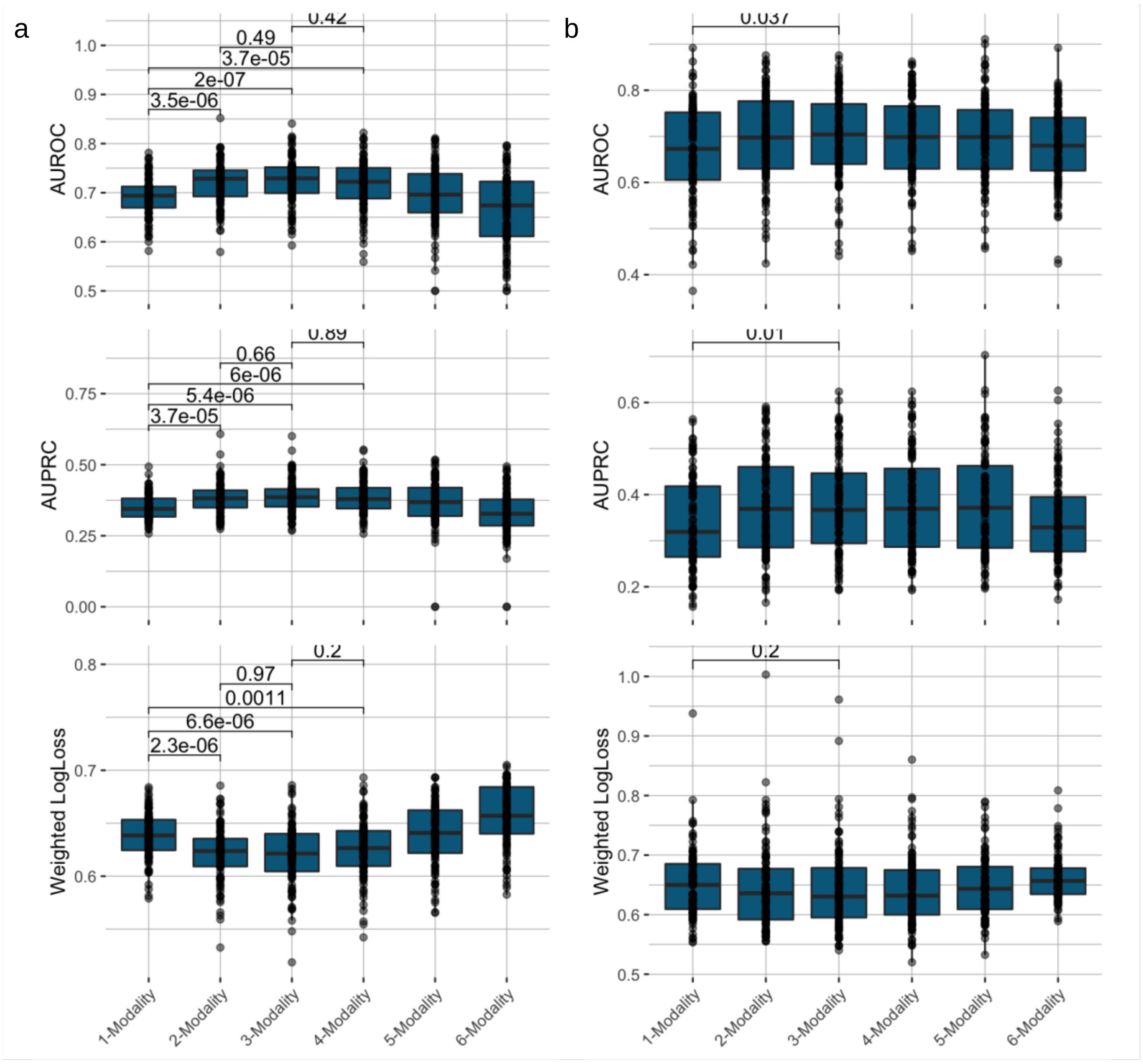

Supplementary Figure S15. Prediction performance of incident DSPN models, when allowing the FFS algorithm to choose starting model based on cross-validation.

a. Prediction performance during cross-validation. X-axis shows the increasing model complexity. Y-axis shows the median of performance values across 5-fold cross-validation for AUROC, AUPRC and weighted log-loss
b. Prediction performance on the testing sets. X-axis shows the increasing model complexity. Y-axis shows the performance values on the testing sets for AUROC, AUPRC and weighted log-loss

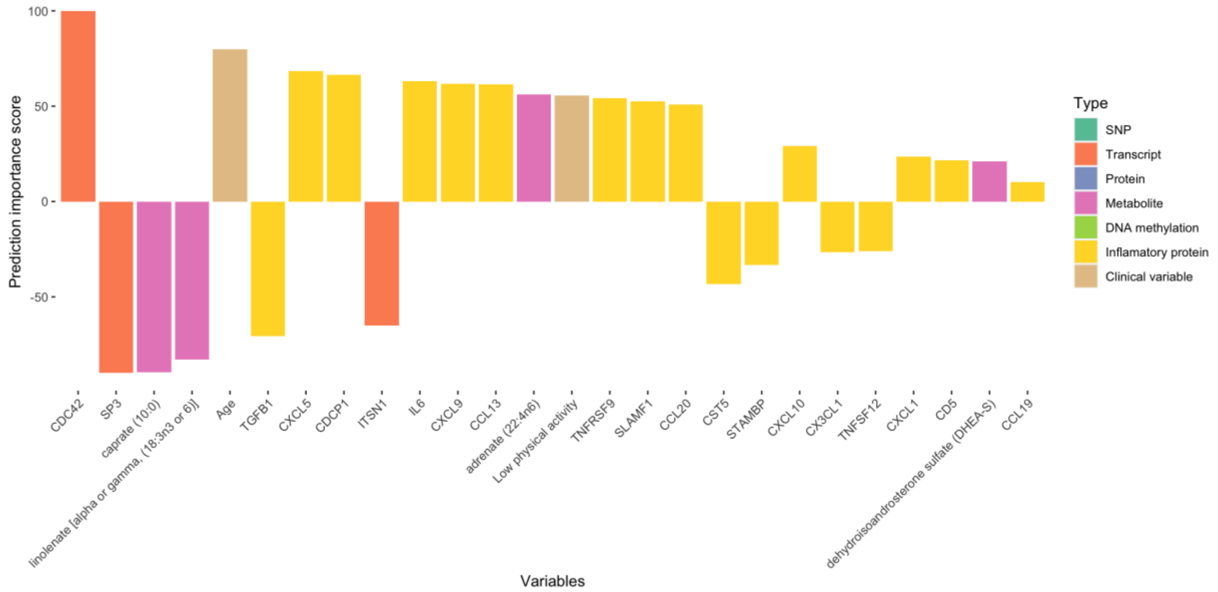

Supplementary Figure S16. Feature importance score of the important features of the final incident DSPN model

X-axis shows the features in decreasing magnitude of the t-statistics in the final model. Y-axis shows the t-statistics (signed importance scores) of the features. Colors represent the data modality.

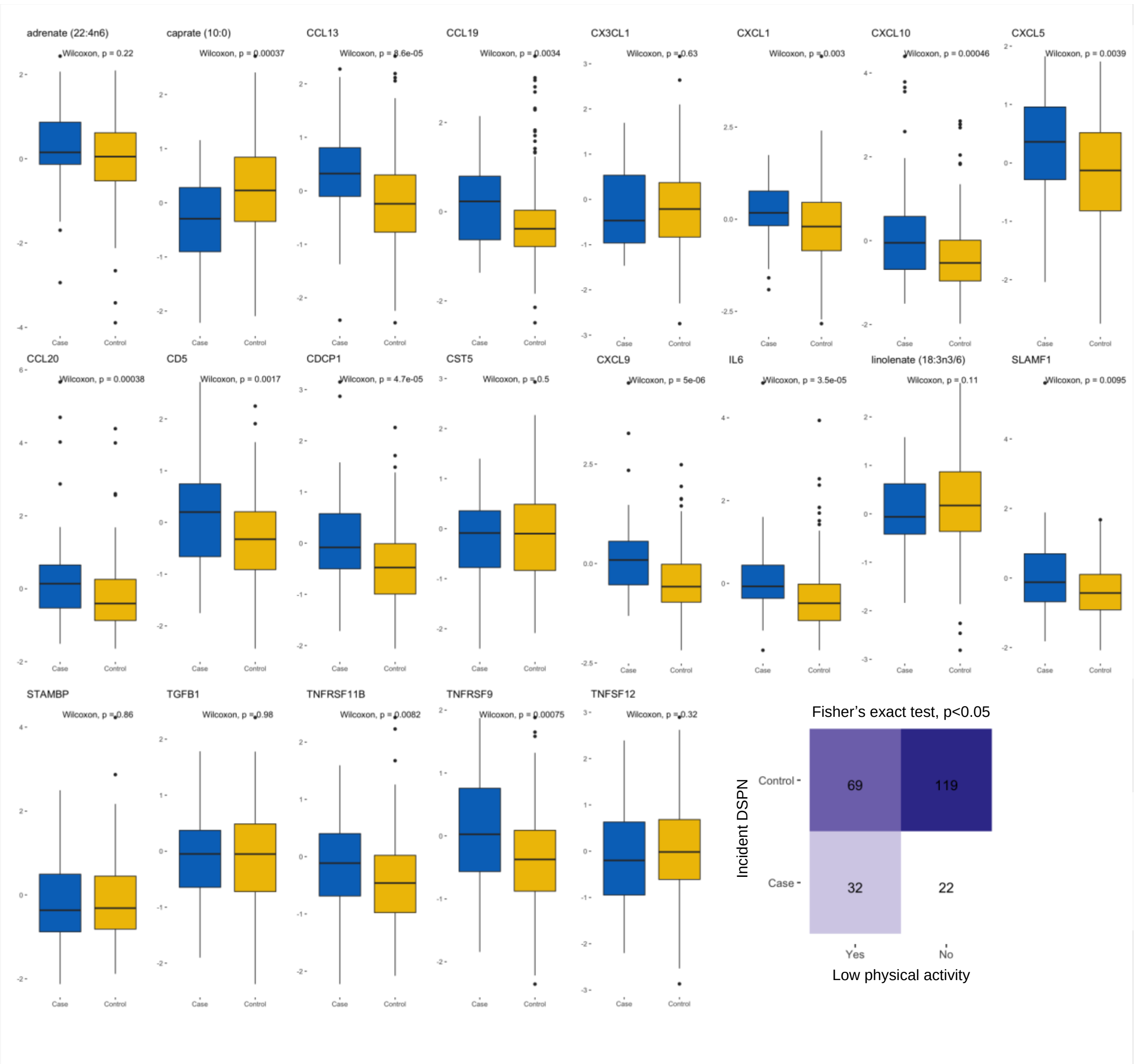

Supplementary Figure S17. Distribution of important clinical variables for incident DSPN model
Distribution of the features in the training set stratified into case and control. P-values for Wilcoxon rank sum test and Fisher's exact test are shown.

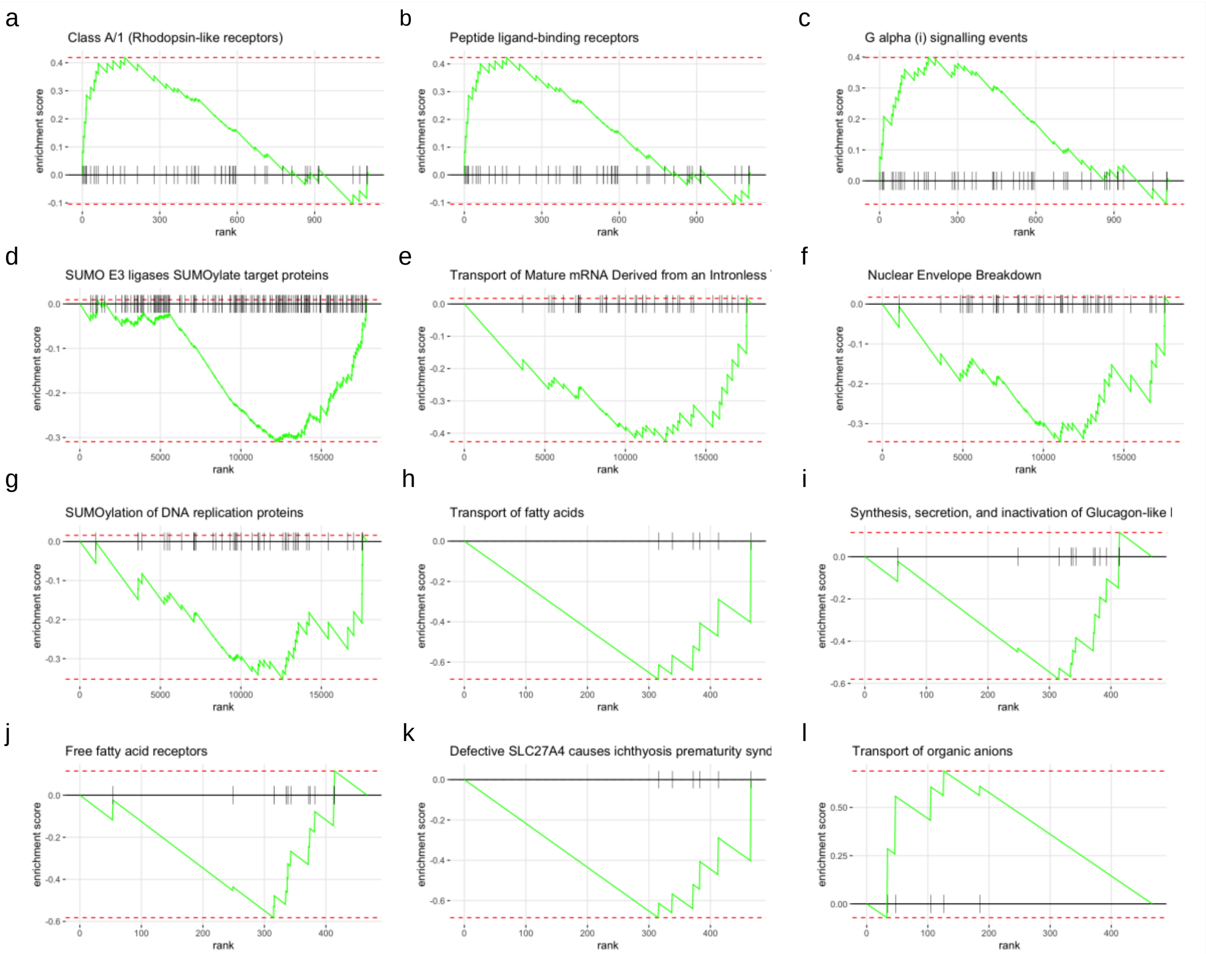

Supplementary Figure S18. Examples of consistently enriched signalling pathways that are predictive of incident DSPN

X-axis represents all evaluated genes ranked in decreasing order of t-statistics, with ticks represent genes that belong to the examined gene set. Y-axis represent the enrichment score. Panels a-c are inflammation protein pathways, d-g are transcriptomic pathways and h-l are metabolomic pathways.

Supplementary Table S1. Clinical characteristics of the dataset for prevalent DSPN prediction

Variable	Control (MNSI <3)	Case (MNSI >= 3)	P
N	903	188	
Age, years	69.7 ± 5.2	72.5 ± 5.2	1.09e-10
Sex, % male	49.4	60.6	0.005
Height, cm	165.3 ± 8.8	167.9 ± 9.6	0.00071
BMI, kg/m2	28.4 ± 4.2	30.2 ± 5.2	1.30e-05
Waist circumference, cm	97.2 ± 11.7	103.7 ± 12.9	8.11e-10
Systolic blood pressure, mmHg	128.8 ± 20	128.6 ± 20	0.873
Diastolic blood pressure, mmHg	74.4 ± 10.1	72.4 ± 9.8	0.007
Hypertension, %	62.0	64.4	0.561
Smoking, %, never/former/current	51.6/40.7/7.7	44.9/48.1/7.0	0.233
High alcohol consumption, %	29.1	33.7	0.220
Low physical activity, %	36.8	51.9	0.014
Previous myocardial infarction, %	5.9	9.1	0.104
Previous stroke, %	3.2	8.0	0.006
Presence of neurological diseases, %	16.2	31.0	4.33e-06
Absent ankle reflexes, %	5	72.3	6.63e-112
Foot ulcer present, %	0	2.1	0.001
MNSI score	1.7 ± 1	4.3 ± 0.9	2.34e-107
Use of NSAIDs, %	3.4	7.4	0.024
NGT, %	53.7	45.7	0.054
i-IFG, %	5.3	3.7	0.464
i-IGT, %	16.7	12.2	0.154
IFG/IGT, %	4.3	6.9	0.133
Newly diagnosed diabetes, %	6.4	4.8	0.504
Known diabetes, %	13.5	26.6	1.25e-05
Diabetes duration, years*	8.1 ± 6.4	15 ± 10.6	1.58e-15
Metabolic parameters			
Fasting glucose, mg/dL[+]	103.6 ± 21.2	110.4 ± 29.9	0.015
2-h glucose, mg/dL[+]	128.0 ± 41.9	127.2 ± 38.6	0.945
HbA1c, %	5.7 ± 0.7	6.0 ± 0.8	3.06e-06
Total cholesterol, mg/dL	222.7 ± 41.0	210.8 ± 37.9	0.00014
LDL cholesterol, mg/dL	140.7 ± 36.2	131.7 ± 33.4	0.001
HDL cholesterol, mg/dL	56.0 ± 14.3	53.4 ± 12.2	0.075
Creatinine, mg/dL	0.95 ± 0.3	1.02 ± 0.3	0.001
Uric acid, mg/dL	5.5 ± 1.4	5.8 ± 1.5	0.015

* Only applicable to people with diabetes

+ Only applicable to people without known diabetes

Supplementary Table S2. Clinical characteristics of the dataset for incident DSPN prediction

Variable	Control (no incident F4->FF4)	Case (incident F4-> FF4)	P
N	394	131	
Age, years	68.0 ± 4.6	70.1 ± 4.9	2.46e-05
Sex, % male	49.2	56.5	0.159
Height, cm	165.9 ± 8.5	167.6 ± 9.4	0.064
BMI, kg/m2	27.7 ± 3.8	29.1 ± 4.0	0.00054
Waist circumference, cm	94.8 ± 11.2	99.9 ± 11.4	1.34e-05
Systolic blood pressure, mmHg	128.4 ± 19.2	131.3 ± 19.9	0.217
Diastolic blood pressure, mmHg	75.5 ± 10.1	75.5 ± 9.2	0.950
Hypertension, %	56.3	65.6	0.066
Smoking, %, never/former/current	52.0/42.4/5.6	55.0/33.6/11.4	0.054
High alcohol consumption, %	29.4	35.9	0.191
Low physical activity, %	26.4	42.7	0.00064
Previous myocardial infarction, %	4.8	6.9	0.373
Previous stroke, %	1.0	0.8	1
Presence of neurological diseases, %	14.7	21.4	0.102
Absent ankle reflexes, %	3.8	6.1	0.323
Foot ulcer present, %	0	0	1
MNSI score	1.5 ± 1.0	1.9 ± 0.9	2.65e-05
Use of NSAIDs, %	1.0	2.3	0.374
NGT, %	62.9	50.4	0.013
i-IFG, %	3.0	7.6	0.040
i-IGT, %	14.5	16.8	0.573
IFG/IGT, %	4.6	4.6	1
Newly diagnosed diabetes, %	5.6	5.3	1
Known diabetes, %	9.4	15.3	0.074
Diabetes duration, years*	6.9 ± 5.5	8.9 ± 5.2	0.116
Metabolic parameters			
Fasting glucose, mg/dL^{+}	101.0 ± 16.4	103.8 ± 17.2	0.078
2-h glucose, mg/dL^{+}	123.9 ± 38.6	127.4 ± 38.4	0.371
HbA1c, %	5.7 ± 0.5	5.8 ± 0.7	0.027
Total cholesterol, mg/dL	226.3 ± 40.5	216.1 ± 42.7	0.009
LDL cholesterol, mg/dL	142.5 ± 36.3	136.6 ± 37.5	0.069
HDL cholesterol, mg/dL	57.3 ± 14.2	52.5 ± 12.3	0.00025
Creatinine, mg/dL	0.9 ± 0.2	1.0 ± 0.3	0.071
Uric acid, mg/dL	5.5 ± 1.3	5.6 ± 1.4	0.692

* Only applicable to people with diabetes

\+ Only applicable to people without known diabetes

Supplementary Table S3. Significantly enriched signalling pathways during feature selection for prevalent DSPN prediction

pathway	pval	padj	ES	NES	size	Type
Formation of a pool of free 40S subunits	1.874e-06	2.024e-05	-0.445	-2.140	95	Transcriptomics
GTP hydrolysis and joining of the 60S ribosomal subunit	3.308e-05	8.132e-05	-0.405	-1.986	104	Transcriptomics
L13a-mediated translational silencing of Ceruloplasmin expression	3.555e-05	8.132e-05	-0.404	-1.972	103	Transcriptomics
Nonsense Mediated Decay (NMD) enhanced by the Exon Junction Complex (EJC)	6.2356e-05	0.0001	-0.395	-1.944	106	Transcriptomics
Collagen chain trimerization	0.0001	0.0002	0.522	2.029	39	Transcriptomics
Influenza Infection	0.0002	0.0004	-0.341	-1.762	143	Transcriptomics
Collagen biosynthesis and modifying enzymes	0.0003	0.0005	0.441	1.921	61	Transcriptomics
Assembly of collagen fibrils and other multimeric structures	0.0003	0.0005	0.448	1.928	58	Transcriptomics
Degradation of the extracellular matrix	0.0004	0.0006	0.338	1.678	131	Transcriptomics
Formation of the ternary complex and subsequently the 43S complex	0.0005	0.0007	-0.460	-1.941	47	Transcriptomics
SUMOylation of DNA methylation proteins	0.0015	0.002	-0.635	-2.006	15	Transcriptomics
Selenoamino acid metabolism	0.0018	0.002	-0.333	-1.642	110	Transcriptomics
Major pathway of rRNA processing in the nucleolus and cytosol	0.0019	0.002	-0.293	-1.550	170	Transcriptomics
Ribosomal scanning and start codon recognition	0.002	0.003	-0.413	-1.774	53	Transcriptomics
Regulation of expression of SLITs and ROBOs	0.005	0.005	-0.291	-1.526	159	Transcriptomics
Laminin interactions	0.0001	0.0713	0.829	2.017	9	Proteomics
Antimicrobial peptides	0.0004	0.0713	-0.676	-2.111	16	Proteomics
Interleukin-20 family signaling	0.0004	0.0713	0.745	2.009	13	Proteomics
Interleukin-3 Interleukin-5 and GM-CSF signaling	0.0006	0.082	0.618	1.922	23	Proteomics
Transport of nucleosides and free purine and pyrimidine bases across the plasma membrane	0.0002	0.0002	-0.931	-2.083	5	Metabolomics
