# Supplementary material for: Interpretable multimodal machine learning (IMML) framework reveals pathological signatures of distal sensorimotor polyneuropathy
Source: Commun Med (Lond). 2024 Dec 16;4:265. doi: 10.1038/s43856-024-00637-1 (PMC11649904; doi:10.1038/s43856-024-00637-1)
Supplement: Supplementary file 3 — Description of Additional Supplementary Files [file 43856_2024_637_MOESM3_ESM.pdf]

Supplementary Data 1. Gene set enrichment analysis result for incident DSPN prediction.

Supplementary Data 2. Reactome gene sets used in GSEA during feature selection by IMML File.

Supplementary Data 3. Data of the results shown in the main figures.
