# Supplementary material for: Interpretable multimodal machine learning (IMML) framework reveals pathological signatures of distal sensorimotor polyneuropathy
Source: Commun Med (Lond). 2024 Dec 16;4:265. doi: 10.1038/s43856-024-00637-1 (PMC11649904; doi:10.1038/s43856-024-00637-1)
Supplement: Supplementary file 7 — Reporting Summary [file 43856_2024_637_MOESM7_ESM.pdf]

nature portfolio

Corresponding author(s): DAPR COMMSMED-23-0324B

Last updated by author(s): Aug 2, 2024

Reporting Summary

Nature Portfolio wishes to improve the reproducibility of the work that we publish. This form provides structure for consistency and transparency in reporting. For further information on Nature Portfolio policies, see our Editorial Policies and the Editorial Policy Checklist.

Statistics

For all statistical analyses, confirm that the following items are present in the figure legend, table legend, main text, or Methods section.

n/a	Confirmed	
☐	☒	The exact sample size (*n*) for each experimental group/condition, given as a discrete number and unit of measurement
☐	☒	A statement on whether measurements were taken from distinct samples or whether the same sample was measured repeatedly
☐	☒	The statistical test(s) used AND whether they are one- or two-sided<br>*Only common tests should be described solely by name; describe more complex techniques in the Methods section.*
☐	☒	A description of all covariates tested
☐	☒	A description of any assumptions or corrections, such as tests of normality and adjustment for multiple comparisons
☐	☒	A full description of the statistical parameters including central tendency (e.g. means) or other basic estimates (e.g. regression coefficient) AND variation (e.g. standard deviation) or associated estimates of uncertainty (e.g. confidence intervals)
☐	☒	For null hypothesis testing, the test statistic (e.g. *F*, *t*, *r*) with confidence intervals, effect sizes, degrees of freedom and *P* value noted<br>*Give P values as exact values whenever suitable.*
☒	☐	For Bayesian analysis, information on the choice of priors and Markov chain Monte Carlo settings
☒	☐	For hierarchical and complex designs, identification of the appropriate level for tests and full reporting of outcomes
☒	☐	Estimates of effect sizes (e.g. Cohen's *d*, Pearson's *r*), indicating how they were calculated

Our web collection on statistics for biologists contains articles on many of the points above.

Software and code

Policy information about availability of computer code

Data collection	No software were used
Data analysis	All code and softwares are available at: https://github.com/phngbh/DSPN

For manuscripts utilizing custom algorithms or software that are central to the research but not yet described in published literature, software must be made available to editors and reviewers. We strongly encourage code deposition in a community repository (e.g. GitHub). See the Nature Portfolio guidelines for submitting code & software for further information.

Data

Policy information about availability of data

All manuscripts must include a data availability statement. This statement should provide the following information, where applicable:

- Accession codes, unique identifiers, or web links for publicly available datasets
- A description of any restrictions on data availability
- For clinical datasets or third party data, please ensure that the statement adheres to our policy

The KORA F4/FF4 cohort dataset is not publicly available due to data protection agreement. However, data access for research purposes could be requested via the KORA platform at https://helmholtz-muenchen.managed-otrs.com/external/.

Research involving human participants, their data, or biological material

Policy information about studies with human participants or human data. See also policy information about sex, gender (identity/presentation), and sexual orientation and race, ethnicity and racism.

Reporting on sex and gender	The findings could be applied to both sexes and genders. In our analysis, sexes and genders were included as a covariate in our statistical model.
Reporting on race, ethnicity, or other socially relevant groupings	The population-based data in this study was obtained from the "Kooperative Gesundheitsforschung in der Region Augsburg/ Cooperative Health Research in the Region of Augsburg" (KORA) platform, which surveyed a homogenous population in the small area of Augsburg.
Population characteristics	See the manuscript's supplementary tables for full description of population characteristics. All relevant variables were included and adjusted for in our analysis
Recruitment	The population-based data in this study was obtained from the "Kooperative Gesundheitsforschung in der Region Augsburg/ Cooperative Health Research in the Region of Augsburg" (KORA) platform 24,45. Specifically, data from the KORA F4 (2006-2008) and the KORA FF4 (2013-2014) studies, both follow-up examinations of the population-based KORA S4 study (1999-2001), were used. Initially there were 1,161 KORA F4 participants aged 62-81 years in the age group with the neuropathy examination module. We excluded 28 individuals with known type 1 diabetes, diabetes forms other than type 2, or unclear glucose tolerance status. In total, we leveraged 1,133 individuals.
Ethics oversight	All examinations were carried out in accordance with the Declaration of Helsinki, including written informed consent from all participants. The study was approved by the ethics board of the Bavarian Chamber of Physicians (Munich, Germany).

Note that full information on the approval of the study protocol must also be provided in the manuscript.

Field-specific reporting

Please select the one below that is the best fit for your research. If you are not sure, read the appropriate sections before making your selection.

☒ Life sciences ☐ Behavioural & social sciences ☐ Ecological, evolutionary & environmental sciences

For a reference copy of the document with all sections, see nature.com/documents/nr-reporting-summary-flat.pdf

Life sciences study design

All studies must disclose on these points even when the disclosure is negative.

Sample size	The population-based data in this study was obtained from the "Kooperative Gesundheitsforschung in der Region Augsburg/Cooperative Health Research in the Region of Augsburg" (KORA) platform 24,45. Specifically, data from the KORA F4 (2006-2008) and the KORA FF4 (2013-2014) studies, both follow-up examinations of the population-based KORA S4 study (1999-2001), were used. Initially there were 1,161 KORA F4 participants aged 62-81 years in the age group with the neuropathy examination module. We excluded 28 individuals with known type 1 diabetes, diabetes forms other than type 2, or unclear glucose tolerance status. In total, we leveraged 1,133 individuals.
Data exclusions	Initially there were 1,161 KORA F4 participants aged 62-81 years in the age group with the neuropathy examination module. We excluded 28 individuals with known type 1 diabetes, diabetes forms other than type 2, or unclear glucose tolerance status. In total, we leveraged 1,133 individuals.
Replication	To ensure the reproducibility of the findings we performed rigorous cross-validations and resamplings wherever possible. Due to the unique design of the prospective dataset, replication with an independent dataset is not yet feasible.
Randomization	Not relevant since this is a case-control observational data
Blinding	Not relevant since this is a case-control observational data

Reporting for specific materials, systems and methods

We require information from authors about some types of materials, experimental systems and methods used in many studies. Here, indicate whether each material, system or method listed is relevant to your study. If you are not sure if a list item applies to your research, read the appropriate section before selecting a response.

Materials & experimental systems

n/a	Involved in the study
☒	☐ Antibodies
☒	☐ Eukaryotic cell lines
☒	☐ Palaeontology and archaeology
☒	☐ Animals and other organisms
☒	☐ Clinical data
☒	☐ Dual use research of concern
☒	☐ Plants

Methods

n/a	Involved in the study
☒	☐ ChIP-seq
☒	☐ Flow cytometry
☒	☐ MRI-based neuroimaging

Plants

Seed stocks	N/A
Novel plant genotypes	N/A
Authentication	N/A
